# Incidence and risk factors for anastomotic leakage after transanal total mesorectal excision in a retrospective cohort of 212 patients

**DOI:** 10.1038/s41598-026-40735-9

**Published:** 2026-03-04

**Authors:** Bartosz Kapturkiewicz, Michal Kazanowski, Pawel Lesiak, David Ramsey, Marek Bebenek

**Affiliations:** 1 Department of Surgical Oncology, Lower Silesian Oncology, Pulmonology and Hematology Center, Pl. Hirszfelda 12, 53-413 Wroclaw, Poland; 2 Department of Oncology and Haematology, Faculty of Medicine, Wroclaw University of Science and Technology, Wroclaw, Poland; 3 Department of Computer Science and Systems Engineering, Wrocław University of Science and Technology, Wroclaw, Poland

**Keywords:** Cancer, Gastroenterology, Oncology, Risk factors

## Abstract

Anastomotic leakage remains a major complication in colorectal surgery. Although several risk factors have been identified, the specific risks associated with TaTME procedures require further clarification. The aim of this study was to determine the frequency of anastomotic leakage after TaTME and to identify factors influencing leakage rates. Out of 237 patients who underwent TaTME, 229 received an anastomosis. Seventeen were excluded from further analysis—14 due to lack of leakage assessment before ileostomy closure and 3 due to missing follow-up data—resulting in a final cohort of 212 patients. Cases were analysed with respect to anastomotic technique and other variables potentially affecting the incidence of anastomotic leakage. Data were obtained from a prospectively maintained institutional database. The mean tumour distance from the anorectal junction (ARJ) was 2.92 cm (± 1.56). Anastomotic leakage occurred in 27 patients (12.74%). The only statistically significant risk factor for leakage was the type of anastomosis: leakage occurred in 18.28% of patients with hand-sewn anastomosis and in 8.47% of those with stapled anastomosis. Tumour height indirectly influenced the leakage rate, as hand-sewn anastomosis was used predominantly in lower tumours (1.78 cm vs. 3.82 cm from the ARJ). Anastomotic leakage rates after TaTME are comparable to those reported for other rectal cancer surgical techniques. Leakage risk is primarily determined by the type of anastomosis and, indirectly, by tumour height. TaTME appears to be a feasible option for selected patients in experienced centres, although further validation is required.

## Introduction

The treatment of rectal cancer has undergone significant transformations over recent decades. Clinical trials have prompted updates in international guidelines regarding both neoadjuvant and adjuvant therapies, as well as surgical approaches. The introduction of Total Mesorectal Excision (TME) by Heald significantly reduced local recurrences and improved 5-year survival rates ^1^. Laparoscopic access provided further benefits, including reduced postoperative pain (particularly opioid use) and accelerated recovery ^2^. However, studies have failed to demonstrate the superiority of laparoscopic access in terms of oncologic outcomes, such as overall survival rates or disease-free survival ^3^. Treating low rectal tumors presents particular challenges due to a restricted operating field, poor visualization of the distal margin, and limited instrument articulation, especially in obese patients. These limitations led John Marks to propose the Transanal Transabdominal Approach (TATA) in 1984, which emphasized controlled achievement of a clear distal margin below the rectal tumor ^4^. In 2010, the Transanal Total Mesorectal Excision (TaTME) surgical technique, the so-called Cecil approach, was introduced by Antonio Lacy’s team, combined transanal and laparoscopic approaches, offering new possibilities for the surgical management of low rectal tumors ^5^. This group of patients, previously often limited to abdominoperineal resection (APR), suddenly had the option of TaTME, enabling preservation of the sphincter and a natural intestinal passage ^6^. Although TaTME offers many benefits for patients, it is not without drawbacks. The incidence of anastomotic leakage is a possible complication of particular significance in such patients, who require ultra-low anastomosis due to mid-to-distal rectal tumors. Large registry data confirm that anastomotic failure remains one of the major concerns after TaTME, with leakage rates of approximately 15% reported in the International TaTME Registry ^7^.

### Objectives

1. To determine the frequency of anastomotic leakage in patients undergoing TaTME surgery.
2. To identify risk factors associated with anastomotic leakage after TaTME and evaluate their impact on leakage rates.

## Methods

### Characteristics of the studied patient group

**Fig. 1:**
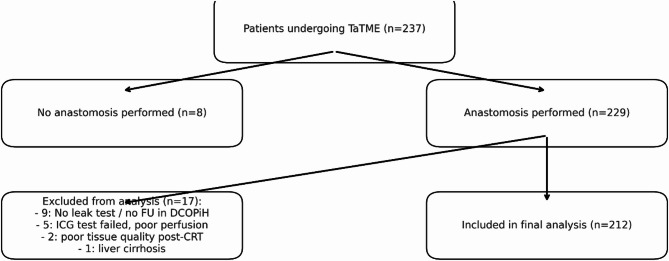
CONSORT-style diagram showing the flow of patients through the study. Exclusion reasons: 8 patients did not receive an anastomosis (5 due to ICG-perfusion failure, 2 due to post-neoadjuvant tissue fibrosis, 1 due to liver cirrhosis); 17 were excluded from leakage analysis (14 had no leak test before ileostomy closure, 3 lacked follow-up data).

The TaTME procedure was first performed at the Lower Silesian Oncology, Pulmonology, and Hematology Center (LSOPHC) in May 2016. Between then and September 2024, 237 patients (165 men and 72 women) with low rectal tumors underwent surgery. From the total of 237 patients who underwent TaTME surgery, 8 were excluded due to inability to perform an anastomosis (5 due to inadequate perfusion in ICG testing, 2 due to extensive neoadjuvant tissue damage, and 1 due to advanced liver cirrhosis). Of the 229 patients with anastomosis, 17 were excluded from leakage analysis: 14 had no leakage test performed before ileostomy closure, and 3 lacked follow-up data. The final analysis included 212 patients. (Fig. 1.) Although the database was prospectively maintained, this study represents a retrospective observational analysis of the recorded cases.

The table below presents the characteristics of the entire cohort of patients qualified for TaTME surgery (*n* = 237) and the subgroup included in the anastomotic leakage analysis (*n* = 212).

**Table 1:** Descriptive statistics for the patient group by variable (either mean with the standard deviation in brackets, or absolutive frequency with relative frequency in brackets, as appropriate).

Variable	Full cohort (*n* = 237)	Study group
Age	61.07 (10.45)	60.64 (10.51)
Gender		
Male	165 (69.6%)	146 (68.9%)
Female	72 (30.4%)	66 (31.1%)
Operative technique		
Hybrid TaTME	37 (15.6%)	35 (16.5%)
Laparoscopic TaTME	200 (84.4%)	177 (83.5%)
Distance from ARJ	2.92 cm (1.56 cm)	2.92 cm (1.56 cm)
Operation time	200.63 min. (46.02 min.)	200.21 min. (46.53 min.)
ICG perfusion test		
Yes	198 (83.5%)	174 (82.5%)
No	39 (16.5%)	38 (17.5%)
BMI	26.58 kg/m^2^ (4.02 kg/m^2^)	26.51 kg/m^2^ (3.96 kg/m^2^)

### Operative technique

All procedures were performed using the two‑team “Cecil” approach originally described by Lacy et al., with both abdominal and transanal teams working concurrently ^8^. Patients were placed in the Lloyd‑Davies lithotomy position with the right arm alongside the body and the left arm on an arm board ^8^. A bilateral transversus abdominis plane (TAP) block was administered in all cases, and general anaesthesia was used.

#### Transanal phase

A disposable transanal platform (GelPOINT^®^) was inserted after circumferential exposure of the rectal lumen ^8^. The transanal team placed a single‑layer purse‑string suture distal to the tumour using 2‑0 Prolene, without performing an intraoperative leak test. Pneumorectum was established at 15 mmHg; the abdominal team worked concurrently with pneumoperitoneum maintained at 12 mmHg until both planes met, at which point pressures were equalised. Continuous smoke evacuation and low‑flow CO₂ insufflation were used to maintain a stable field of vision ^8^. After a full‑thickness incision of the rectal wall distal to the purse‑string, dissection proceeded in the posterior mesorectal plane and then sequentially along the anterior and lateral planes until the abdominal team’s dissection was reached. This stepwise approach is commonly adopted by experienced TaTME centres ^4,6,8^.

#### Abdominal phase

A five‑port laparoscopic configuration was used: a 10 mm optical trocar was placed slightly to the right of the umbilicus, two 5 mm ports were positioned in the epigastrium and right middle abdomen, and an optional 12 mm port was inserted suprapubically for stapler introduction. The left colon was mobilised using a standard medial‑to‑lateral approach, with high ligation of the inferior mesenteric artery and routine mobilisation of the splenic flexure. The peritoneal reflection was divided anteriorly, and indocyanine green fluorescence angiography was employed from the 40th case to assess bowel perfusion ^6^.

The specimen was extracted through a Pfannenstiel incision. Stapled anastomoses were preferred when at least 2 cm of distal rectal stump allowed placement of the purse‑string suture; a 33 mm circular stapler was used in these cases. A double-stapling technique was not used in any case; all stapled anastomoses were performed with a single circular stapler firing after a transanal purse-string suture, in keeping with standard TaTME practice. Hand‑sewn coloanal anastomoses were performed when stapling was not feasible (usually tumours ≤ 2 cm from the anorectal junction) with a single‑layer interrupted 3‑0 monofilament suture. An air leak test was performed routinely by filling the pelvis with saline and insufflating the rectum; results were documented in the operative record ^8^.

A diverting loop ileostomy was created in more than 95% of cases based on institutional policy and predefined risk factors ^8^. Diversion was omitted only when perfusion and anastomotic integrity were deemed optimal at the end of the procedure ^6^.

Oncologic parameters. Long-term oncologic outcomes (lymph-node yield, margin status, and recurrence patterns) were evaluated descriptively in the first 237 TaTME resections, corresponding to the time cohort for which follow-up data were available. A formal analysis of overall and disease-free survival will be reported in a separate manuscript focused specifically on long-term oncologic results.

### Definition and assessment of anastomotic leakage

Anastomotic leakage was defined in accordance with the International Study Group of Rectal Cancer (ISREC) criteria as a defect of the intestinal wall integrity at the colorectal or coloanal anastomosis leading to a communication between the intra- and extraluminal compartments. This definition included clinical signs of peritonitis, pelvic abscess, feculent discharge from drains, or radiological evidence of anastomotic dehiscence confirmed by contrast-enhanced imaging or digital rectal examination.

Leakage events were classified according to the ISREC grading system as follows:

-  Grade A – asymptomatic leakage not requiring active therapeutic intervention.
-  Grade B – leakage requiring active therapeutic intervention without the need for reoperation (e.g., antibiotics, percutaneous drainage, transanal endoluminal vacuum therapy).
-  Grade C – leakage requiring reoperation.

Because several patients in the institutional database had treatment codes indicating management of leakage despite not being initially marked as “leakage = yes”, the dataset was re-audited prior to analysis. All cases with documented leakage-related intervention (grade B or C), as well as all cases with radiological or clinical evidence of leakage managed conservatively (grade A), were included in the final classification.

This process identified a total of 27 anastomotic leaks: 16 grade A, 4 grade B, and 7 grade C.

To facilitate statistical modelling of leakage severity, an ordinal variable (“Significance of leakage”) with four ordered categories was created:

0 = no leakage,

1 = grade A,

2 = grade B,

3 = grade C.

The qualification criteria for rectal tumor patients at LSOPHC were based on guidelines from Polish and international scientific societies, such as the Polish Society of Clinical Oncology (PSCO), the Polish Society of Surgical Oncology (PSSO), the European Society for Medical Oncology (ESMO), the European Society of Surgical Oncology (ESSO), and the National Comprehensive Cancer Network (NCCN). Multidisciplinary teams assessed patients using colonoscopy, pelvic MRI, chest and abdominal CT, and digital rectal examination by the operating surgeon.

Patients eligible for TaTME underwent additional preoperative sphincter function assessment based on sphincter manometry. Such patients had Rullier classification I-III tumors located 0–6 cm from the anorectal junction (ARJ), mean tumor distance of 2.92 cm from the ARJ. Of these patients, 172 (81.1%) received neoadjuvant therapy. Of the remaining patients, 50 (23.6%) underwent TaTME as a primary treatment, and 15 (7.1%) underwent TaTME as a salvage procedure following incomplete endoscopic tumor resection. Neoadjuvant patients received standalone radiotherapy (RTH; 91 patients), or combined chemoradiotherapy (CHTH; 79 patients) in the form of either concurrent 28-day radiochemotherapy or sequential chemotherapy (3–4 cycles of XELOX or FOLFOX) preceded by short-course RTH (5 × 5 Gy). The remaining 2 patients received standalone chemotherapy (CHTH) as a neoadjuvant treatment.

**Table 2:** Assigned treatment for the entire patient group by the tumor board.

RTH	RTH+CHTH	Local extraction	CHTH	None
91 (38.39%)	79 (33.33%)	15 (6.32%)	2 (0.84%)	50 (21.09%)

Anthropometric data (height, weight) were collected from each patient, enabling calculation of the BMI, with an average BMI of 26.58 kg/m², ranging from 17.75 to 41.28 kg/m². The physical state of patients was assessed using the WHO scale, and perioperative risk according to the ASA classification, with 141 patients rated as WHO grade 0, 96 as grade 1. The ASA scale of risk assessed 11 patients as being in ASA I, 180 in ASA II, 44 in ASA III, and 2 in ASA IV.

Normality of continuous variables was assessed using the Shapiro–Wilk test, and non-parametric alternatives were applied where appropriate. Statistical analysis included the use of Fisher’s exact test, Welch’s t-test, Pearson’s chi-square test, and Bayesian network analysis. To analyse the ordered severity categories of anastomotic leakage (0 = no leakage; 1 = grade A; 2 = grade B; 3 = grade C), an ordinal regression model was constructed. Variables initially entered into the model included sex, frailty status, tumour height (distance from the anorectal junction), duration of surgery, and anastomotic technique (hand-sewn vs. stapled). Non-significant variables were sequentially removed until only predictors contributing significantly to the model remained. A significance threshold of *p* < 0.05 was applied.

## Results

Baseline demographic, clinical, and treatment characteristics of the study cohort are summarised in Tables 1, 2 and 3.

In the initial phase of TaTME surgeries at LSOPHC, a hybrid approach was adopted for logistical reasons, integrating the transanal phase of TaTME with an open abdominal component. This method was applied in 37 surgeries, with open access due to simultaneous liver lesion resections in two cases. After reorganizing the operating room and surgical team, the fully laparoscopic TaTME technique, as described by A. Lacy and colleagues, was adopted. A total of 200 procedures were performed in this manner, with a 3.5% conversion rate to open surgery (7 patients). Beginning with the 40th procedure, intraoperative perfusion assessment using indocyanine green (ICG) fluorescence imaging was introduced. This technique was subsequently applied in 198 cases, accounting for 83.5% of the patient cohort. Among the 237 TaTME patients, intestinal anastomosis was performed in 229, and 8 patients did not receive an anastomosis, representing 96.6% and 3.4% of the total, respectively. In most cases, the lack of anastomosis was due to poor vascular perfusion as indicated by ICG fluorescence, leading to non-anastomotic management in 5 patients. In the remaining group, the absence of anastomosis resulted from severe post-neoadjuvant tissue changes in 2 patients and concerns about impaired anastomotic healing in 1 patient with advanced liver cirrhosis. The choice of the method of anastomosis depended on the tumor’s location: hand-sewn anastomosis was preferred for tumors very close to the ARJ, whereas stapled anastomosis was used for tumors located at least 1–2 cm from the ARJ. Among the 229 patients who received anastomosis, 99 had hand-sewn and 130 had stapled anastomosis, constituting 43% and 57% of all anastomoses, respectively. For mechanical anastomosis, a 33 mm hemorrhoidal stapler was used in 114 cases (87.7%), with other staplers (28–31 mm) being used in the remaining 12.3%. In the analysed group of 229 patients with anastomosis, 218 (95.2%) received protective ileostomies based on predefined risk factors for leakage. In 11 cases (4.8%), an ileostomy was not performed due to intraoperative confirmation of anastomotic integrity. The average duration of a TaTME operation was approximately 200 min, with a median of 195 min and a range from 110 to 365 min.

### Statistical analysis of the study group

Statistical analysis was performed on 212 patients for whom the presence or absence of anastomotic leakage could be definitively established. Anastomotic leakage occurred in 27 patients (12.74%). This rate is consistent with previously published large-scale data, including the International TaTME Registry, which reported an overall anastomotic leakage rate of approximately 15% in 1594 patients^7^. The following clinical and perioperative variables were included in the analysis: age, sex, WHO performance status, ASA classification, BMI, type of preoperative treatment, surgical approach (open vs. laparoscopic), use of indocyanine green perfusion assessment, anastomotic technique (hand-sewn vs. stapled), presence of a protective ileostomy, operative duration, and tumour distance from the anorectal junction. Of the analysed patients, 66 (31.13%) were female and 146 (68.87%) were male. WHO performance status was 0 in 130 patients (61.32%) and 1 in 82 patients (38.68%). ASA physical status was stratified into two groups due to low frequency of extreme grades: low-risk (ASA I–II; 172 patients, 81.13%) and high-risk (ASA III–IV; 40 patients, 18.87%). Preoperative treatment was administered to 167 patients (78.77%).

**Table 3:** Assigned treatment for the patient group subjected to statistical analysis by the tumor board.

RTH	RTH+CHTH	Local extraction	CHTH	None
80 (37.74%)	70 (33.02%)	14 (6.60%)	2 (0.94%)	45 (21.70%)

### Severity of anastomotic leakage

The distribution of anastomotic leakage severity according to patient- and procedure-related factors is presented in Table 4.

According to the ISREC classification, 27 patients (12.74%) developed an anastomotic leak: 16 (59.3%) grade A, 4 (14.8%) grade B, and 7 (25.9%) grade C. Clinically significant leakage (grades B–C) occurred in 11 patients (5.2% of the entire cohort). The distribution of ISREC grades in our cohort is comparable to international datasets, including the International TaTME Registry^7^.

Leakage severity was associated with several clinical variables. More severe leaks (grade C) occurred more frequently in patients classified as not frail (Fisher’s exact test, *p* = 0.0315). Male sex was associated with higher rates of clinically significant leakage (grades B–C) compared with females (*p* = 0.0152).

The type of anastomosis was also significantly associated with severity: hand-sewn anastomoses had a higher proportion of grade C leaks compared with stapled anastomoses (*p* = 0.0121).

Ordinal regression analysis confirmed that the type of anastomosis was the only independent predictor of increasing leakage severity, while sex, frailty, tumour height, and operative time were not significant in the final model.

A summary of severity distribution by sex, frailty, and anastomotic technique is provided below in Table 4:

**Table 4:** Distribution of leakage severity by frailty, sex, and anastomotic technique.

Variable	No leakage	Grade A	Grade B	Grade C	Variable
Not frail	117 (90.0%)	4 (3.1%)	3 (2.3%)	6 (4.6%)	Not frail
Frail	70 (85.4%)	10 (12.2%)	1 (1.2%)	1 (1.2%)	Frail
Female	60 (90.9%)	6 (9.1%)	0 (0%)	0 (0%)	Female
Male	127 (87.0%)	8 (5.5%)	4 (2.7%)	7 (4.8%)	Male
Hand-sewn anastomosis	77 (82.8%)	6 (6.5%)	4 (4.3%)	6 (6.5%)	Hand-sewn anastomosis
Stapled anastomosis	109 (92.4%)	8 (6.8%)	0 (0%)	1 (0.9%)	Stapled anastomosis

Associations between surgical approach, patient risk profiles, perioperative factors, and anastomotic leakage are summarised in Tables 6, 7, 8, 9, 10 and 11.

ICG scanning was used for 174 patients (82.46%). One patient underwent a Hartmann procedure and was excluded from further analysis. In the remaining operations, hand-sewn anastomosis was used in 93 operations (44.08%) and stapled anastomosis in 118 cases (55.92%). A hemorrhoidal stapler was used in 104 cases (88.1%), and a rotational stapler was used in 14 cases (11.9%). Open surgery was carried out in 35 cases (16.59%) and a laparoscope was used in 176 operations (83.41%).

Among the first 237 TaTME resections, the mean lymph‑node yield was 14.4 ± 7.2 nodes (median 13 nodes; one missing value). Positive circumferential radial margins were recorded in 2 cases (~ 0.8%), while distal margin involvement occurred in 3 cases and no proximal margin involvement was documented.

During follow‑up, 12 patients (5.06%) developed local recurrence and 30 patients (12.7%) developed distant metastases. Overall, 34 patients (14.3%) experienced any oncologic event (local or distant).

Mesorectal specimen quality according to the MERCURY/Quirke grading system was available for 126 patients. Among these, 72.7% (*n* = 96) had a complete mesorectal excision (grade I), 18.2% (*n* = 24) had a near-complete excision (grade II), and 4.5% (*n* = 6) had an incomplete excision (grade III).

Taken together, the high rate of complete or near-complete mesorectal excision and the low CRM-positivity rate indicate that oncologic resection quality in this cohort met contemporary standards (Table 5).

**Table 5:** Mesorectal excision quality according to the MERCURY/Quirke grading system (only cases with complete data).

MERCURY grade	Description	*n*	%
I	Complete TME	96	72.7%
II	Near-complete TME	24	18.2%
III	Incomplete TME	6	4.5%
Total	—	126	100%

**Fig. 2:**
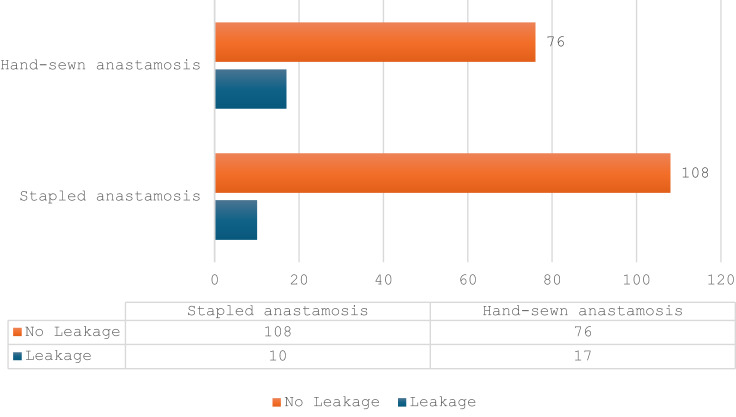
The relationship between the method of stapling and anastomotic leakage.

When analysing the relationship between anastomotic leakage and the other variables, one approach is to use multivariate logistic regression. Tumour height was included as an additional variable in the multivariable model. For analysis, tumour distance from the anorectal junction was dichotomised into ≤ 2 cm and > 2 cm, reflecting the typical threshold for determining feasibility of stapled anastomosis. In the same multivariable logistic regression model, tumour height was also found to be significantly associated with anastomotic leakage. Tumours located ≤ 2 cm from the anorectal junction were associated with an increased risk of leakage (OR = 3.45; 95% CI: 1.51–7.89; *p* = 0.003). Furthermore, tumour height analysed as a continuous variable showed that each additional centimetre above the anorectal junction reduced the odds of leakage by approximately 49% (OR = 0.51; 95% CI: 0.28–0.92; *p* = 0.026). A stepwise procedure was used, such that the initial model contained all the explanatory variables. At each stage, the least significant variable was removed from the model, until all of the remaining variables were significant at the 5% level. In the multivariable logistic regression model including both the type of anastomosis and tumour height, the stapled technique remained a statistically significant protective factor against anastomotic leakage. Compared to stapled anastomosis, hand-sewn anastomosis was associated with an increased risk of leakage (OR = 3.52, 95% CI: 1.07–11.6, *p* = 0.038). Additionally, tumour height was inversely correlated with leakage risk; each additional centimetre of distance from the anorectal junction reduced the odds of leakage by approximately 49% (OR = 0.51, 95% CI: 0.28–0.92, *p* = 0.026). (Fig. 2)

The frequency of leakage when stapled anastomosis is used is low and applies to 10 out of 118 cases, accounting for 8.47%. When hand-sewn anastomosis is used the frequency of leakage is 18.28% and applies to 17 out of 93 cases.

**Fig. 3:**
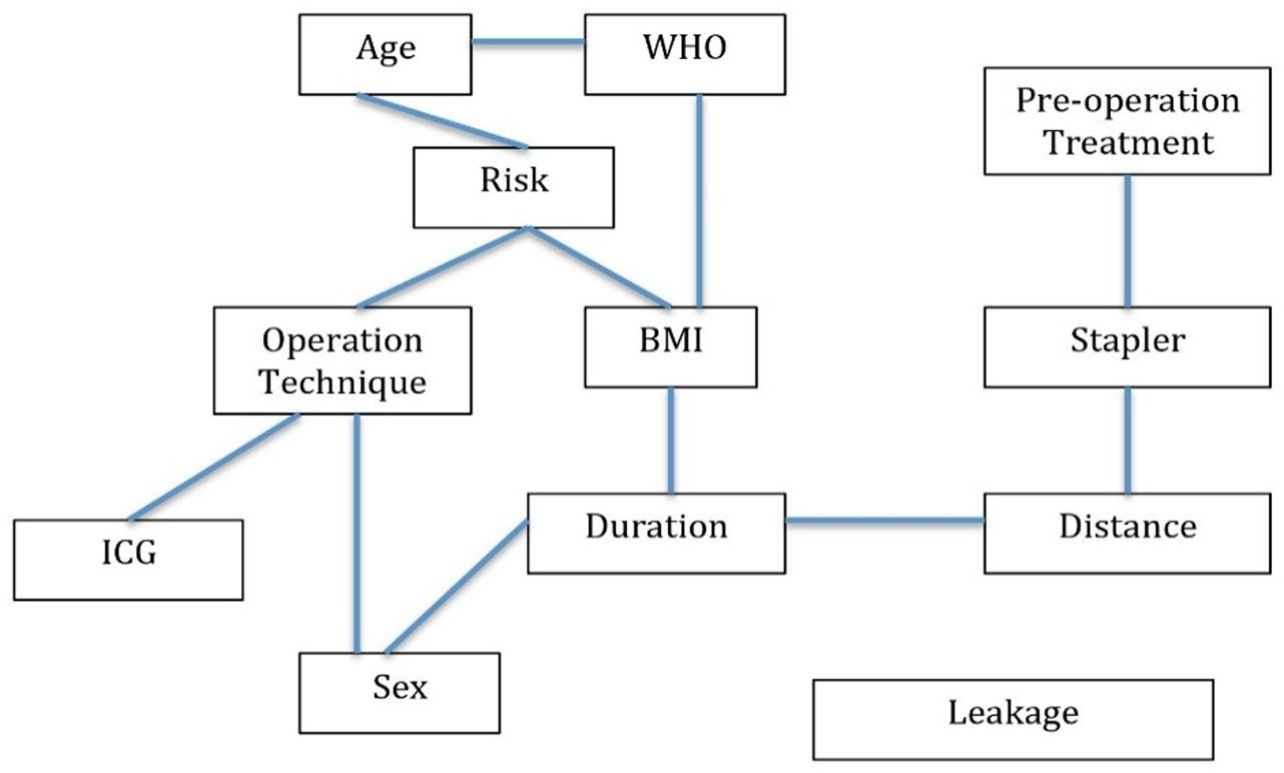
Relationship between the observed variables.

Another approach employed was Bayesian network analysis, using the abn package in R^9^ to explore the structure of relationships among all observed variables. In this model, no direct link was observed between any variable and anastomotic leakage. The inferred network is shown in Fig. 3, with direct dependencies indicated by connecting lines.

This discrepancy between the logistic regression and Bayesian network results likely reflects differences in model assumptions and sensitivity. Bayesian networks identify only direct probabilistic dependencies and are influenced by sample size and effect magnitude. In our study, the association between anastomotic leakage and anastomosis type—although statistically significant in the logistic regression—may have been attenuated in the network model due to collinearity with tumour height or the near-universal use of protective ileostomy. These factors may obscure direct causal paths in a Bayesian graphical model. Although formal multicollinearity diagnostics such as variance inflation factors (VIF) were not calculated, the regression model was constructed using stepwise variable elimination. Stability of the odds ratios across model iterations suggests no major multicollinearity among the included variables. (Fig. 3)

Therefore, the absence of a direct connection in the Bayesian model should not be interpreted as evidence against an association, but rather as a methodological complement that reflects the complexity of the underlying relationships.

The analysis of the relationship between the choice of treatment (e.g. open or laparoscopic) and the other variables observed demonstrates that ICG scanning was almost exclusively used with laparoscopic surgery (Fisher’s exact test of association, *p* ≈ 0). The percentages correspond to the numbers observed in a row.

**Table 6:** Use of indocyanine green in vascular perfusion testing.

	Low risk	High risk
Laparoscopic	149 (84.66%)	27 (15.34%)
Open	22 (62.86%)	13 (37.14%)

**Fig. 4:**
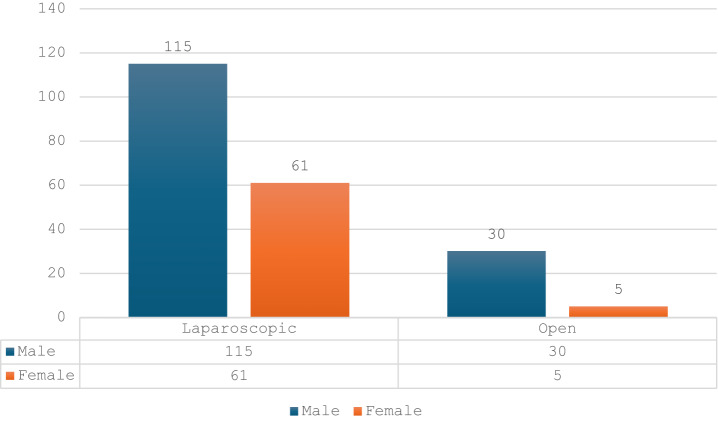
The use of the surgical technique by gender.

Additionally, female patients had a significantly higher likelihood of undergoing laparoscopic surgery compared to males (Fisher’s exact test, *p* = 0.0171). Laparoscopy was performed on 61 women and 115 men (respectively 34.46% and 65.54% laparoscopic cases), whereas the hybrid technique using the open approach was applied to 5 women and 30 men (respectively 14.29% and 85.71% cases of the hybrid approach) (Fig. 4)

Open surgery is used more often when the patient is assessed to be high risk (Fisher’s exact test of association, *p* = 0.007742). The laparoscopic technique was used for 149 (84.66%) low-risk patients and 27 (15.34%) high-risk patients, whereas the open technique was applied to 22 (62.86%) low-risk patients and 13 (37.14%) high-risk patients.

**Table 7:** The use of the surgical technique by ASA risk.

	Low risk	High risk
Laparoscopic	149 (84.66%)	27 (15.34%)
Open	22 (62.86%)	13 (37.14%)

It should be noted that higher BMI was significantly associated with a higher perioperative risk classification (ASA categories 3 and 4). The mean BMI of high risk patients was 28.74 with a standard deviation of 3.43. The mean BMI of low risk patients was 25.99 with a standard deviation of 5.19 (Welch’s t-test, *p* = 0.002572). A similar association was observed between BMI and WHO performance status. The mean BMI of patients in a poor state according to WHO was 27.67 with a standard deviation of 3.51. The mean BMI of patients in a good state was 25.78 with a standard deviation of 4.36 (Welch’s t-test, p-value = 0.001167).

When analysing the relationship between the type of anastomosis and other variables no significant association was found between stapling technique and surgical approach (Fisher’s exact test, *p* = 0.197). There is a clear association between the use of a stapled anastomosis and the distance between the tumor and the anorectal junction (Welch’s t-test, *p* ≈ 0). Amongst the patients that underwent hand-sewn anastomosis this mean distance was 1.78 cm with a standard deviation of 1.18 cm. Amongst the patients that underwent surgery with a stapled anastomosis this mean distance was 3.82 cm with a standard deviation of 1.20 cm.

Stapled anastomosis was more likely to be used when the patient had received some form of pre-operation treatment (Fisher’s exact test of association, *p* = 0.01078). In the group of patients who did not receive preoperative treatment, 27 underwent hand-sewn anastomosis, while 17 underwent stapled anastomosis. Similarly, in the group of patients who received neoadjuvant treatment, hand-sewn anastomosis was performed in 66 cases and stapled anastomosis was performed in 101 cases.

Neoadjuvant therapy was more frequently administered in patients with tumors further from the anorectal junction. Among those who did not receive pre-operational treatment, the mean distance was 2.44 cm with a standard deviation of 1.50 cm. Among those that received pre-operational treatment, the mean distance was 3.05 cm with a standard deviation of 1.56 cm (Welch’s t-test, *p* = 0.02069).

Patients in a good physical state according to the WHO are, on average, significantly younger (mean 57.30, std. dev. 10.40) than those who are assessed to be in a poor state (mean 65.74, std. dev. 8.42, Welch’s t-test, *p* ≈ 0). Similarly, younger patients are more likely to be assigned to a low risk group than older patients. The mean age of patients in risk groups 1 and 2 was 58.94 with a standard deviation of 10.53. The mean age of patients in risk groups 3 and 4 was 67.63 with a standard deviation of 6.94 (Welch’s t-test, *p* ≈ 0).

The length of an operation is associated with a number of variables. There is a relatively large positive correlation (*r* = 0.3988) between a patient’s BMI and the length of an operation (Pearson’s test of association, *p* ≈ 0) and the use of ileostomy is associated with lengthy operations. When no ileostomy was used, the mean duration of an operation was 148.75 min. with a standard deviation of 19.41 min. When ileostomy was used, the mean duration of an operation was 202.09 min. with a standard deviation of 46.21 min (Welch’s t-test, *p* = 0.0000285). It should be noted that the use of ileostomy was also positively associated with the BMI of a patient. In addition, the average length of an operation was associated with sex. For operations on males, the mean duration was 207.03 min. with a standard deviation of 47.65 min. For operations on females, the mean duration was 184.77 min. with a standard deviation of 40.47 min (Welch’s t-test, *p* = 0.0006185). The length of an operation was also significantly positively correlated with the age of a patient (*r* = 0.1434, Pearson’s test of association, *p* = 0.03737). The length of an operation was negatively associated with the distance between the tumor and the anorectal junction. However, this association is not significant (*r*=−0.1313, Pearson’s test of association, *p* = 0.05684).

In a logistic regression model, the type of anastomosis was significantly associated with the risk of anastomotic leakage. The odds of leakage were lower in patients with stapled anastomoses compared to those with hand-sewn anastomoses (OR = 0.41; 95% CI: 0.17–0.99; *p* = 0.0383). This effect remained significant even after including tumour height in the multivariable model, supporting stapled anastomosis as an independent protective factor. Propensity score methods were not used, as tumour height—identified as a potential confounder—was directly included in the multivariable logistic regression model.

There is no significant association between the form of pre-operation treatment used and the frequency of leakage (ignoring the 2 cases in which CHTH was used alone).

**Table 8:** Association between pre-operation treatment and anastomosis leakage. Note: Two patients who received standalone CHTH were excluded from this comparison due to small sample size. The frequency of leakage is greater when a laparoscope was used than when open surgery was carried out. However, the number of open surgeries was relatively small and so this difference is not significant.

	No leakage	Leakage
None	40 (86.96%)	6 (13.04%)
RTH	67 (83.75%)	13 (16.25%)
RTH+CHTH	64 (91.43%)	6 (8.57%)
Local extraction	13 (92.86%)	1 (7.14%)

**Table 9:** Association between type of surgical procedure and anastomosis leakage. When analysing the frequency of leakage according to the state of the patient, it was noted that the frequency of leakage is around 10–15% independently of the physical state of the patient according to WHO (Fisher’s exact test of association, *p* = 0.5312).

	No leakage	Leakage
Open	32 (91.43%)	3 (8.57%)
Laparoscope	153 (86.44%)	24 (13.56%)

**Table 10:** Association between physical capacity according to the WHO scale and anastomosis leakage. Similarly, there is no significant association between leakage and the assessed level of risk (Fisher’s exact test of association, *p* = 0.3024).

	No leakage	Leakage
WHO state good	115 (88.46%)	15 (11.54%)
WHO state bad	70 (85.37%)	12 (14.63%)

**Table 11:** Association between perioperative risk according to the ASA scale and anastomosis leakage.

	No leakage	Leakage
Low risk	152 (88.37%)	20 (11.63%)
High risk	33 (82.50%)	7 (17.50%)

## Discussion

In our series, the anastomotic leakage rate of 12.74% falls within the range reported in contemporary literature and is likely influenced by the high proportion of patients with very low rectal tumours, a recognised risk factor for leakage^7,10–12^. Similar leakage rates have been reported in recent multicentre randomised studies evaluating transanal total mesorectal excision (TaTME) for mid- and low-rectal cancer^7,10,13,14^.

In our cohort, hand-sewn anastomosis was associated with a higher rate of anastomotic leakage than stapled anastomosis (18.28% vs. 8.47%; *p* = 0.0383). As the choice of technique was determined by tumour height and anatomical feasibility, stapled anastomosis was used when sufficient distal rectal length allowed secure purse-string placement, whereas hand-sewn anastomosis was reserved for the most distal, technically challenging tumours. In the multivariable model, both anastomotic technique and tumour height remained independent predictors of leakage, supporting the interpretation that the increased risk observed after hand-sewn anastomosis primarily reflects case selection in ultra-low tumours rather than an intrinsic limitation of the technique^10–12,15^.

Multivariable logistic regression confirmed that anastomotic technique remained an independent predictor of leakage after adjustment for tumour height, supporting the interpretation that stapled anastomosis, when anatomically feasible, is associated with a lower leakage risk. These findings are consistent with evidence linking lower tumour position to increased technical complexity and postoperative morbidity in rectal surgery^10–12^, as well as with recent Bayesian network meta-analyses and multicentre randomised trials reporting comparable histopathological quality and complication rates between transanal, laparoscopic, robotic, and open approaches^14,16^.

Exploratory analysis of the hand-sewn subgroup showed that these anastomoses were performed almost exclusively in patients with ultra-low rectal tumours (mean height 1.8 cm from the ARJ), in whom stapled anastomosis was anatomically not feasible. Baseline characteristics, including age, BMI, ASA class, tumour stage and neoadjuvant treatment, were comparable between stapled and hand-sewn groups, supporting the interpretation that the higher leakage rate observed after hand-sewn anastomosis reflects case selection and anatomical difficulty rather than a technique-specific limitation. All hand-sewn anastomoses were protected with a diverting ileostomy, and oncologic parameters (CRM status, lymph-node harvest and recurrence patterns) were comparable between groups.

Neoadjuvant treatment was not associated with the incidence of anastomotic leakage, consistent with observations from other studies^10,11,17^. Demographic and clinical variables, including age, sex, BMI, tumour stage, performance status and ASA classification, were also not associated with leakage risk. A diverting ileostomy was created in 95% of patients, reflecting routine practice in ultra-low rectal surgery; therefore, its impact on the clinical manifestation of leakage could not be assessed in this cohort.

Although several clinical factors may influence operative planning, in our practice anastomotic technique was primarily determined by tumour height, whereas surgical approach was largely influenced by operating room logistics. The impact of these variables on operative duration will be analysed in a separate study. Our findings are consistent with recent European multicentre propensity score–matched analyses comparing TaTME with laparoscopic and robotic techniques in locally advanced rectal cancer^18^.

Descriptive oncologic outcomes from our cohort support the oncologic adequacy of TaTME in our centre. A mean lymph-node yield of approximately 14 and a circumferential radial margin positivity rate below 1% compare favourably with published series. Among the first 237 patients, local recurrence occurred in 5.06% and distant metastases in 12.7%, both within the expected range reported for mid- and low-rectal cancer despite the technical complexity of ultra-low tumours. MERCURY grading further confirmed high-quality TME performance, with over 90% complete or near-complete mesorectal excisions. Detailed analyses of overall and disease-free survival and multivariable predictors of oncologic outcomes will be reported separately.

This study has several limitations related to its retrospective design and single-centre setting. Incomplete and non-uniform clinical documentation limited the precise reconstruction of symptom severity and timing of individual leakage events, despite detailed re-audit and ISREC A/B/C grading. Confounding by indication must be acknowledged, as the choice between stapled and hand-sewn anastomosis was dictated by tumour height and anatomical constraints rather than randomisation, and therefore reflects the technical complexity of ultra-low tumours^12,15^. The near-universal use of diverting ileostomy (~ 95% of patients) limits assessment of the natural history and clinical severity of leakage, as protective diversion may attenuate clinical manifestations and reduce the proportion of grade B–C events. The cohort spans eight years with evolving operative techniques, including transition from hybrid to fully laparoscopic TaTME and adoption of indocyanine-green perfusion; however, the dataset was not powered to evaluate temporal trends. No formal sample-size or power calculation was performed, and with only 27 leakage events, multivariable analyses are at risk of type II error. Finally, as a high-volume single-centre experience, the generalisability of these findings to lower-volume centres may be limited. Although descriptive oncologic outcomes are reported, detailed analyses of overall and disease-free survival lie beyond the scope of this manuscript and will be presented separately.

Standardised MERCURY/Quirke mesorectal grading was implemented part-way through the study period; therefore, mesorectal quality could be assessed in only 126 patients and could not be incorporated into multivariable modelling. Additional statistical limitations should also be acknowledged. The multivariable model was developed using stepwise regression, which carries a recognised risk of overfitting and model instability in moderate-sized datasets. Moreover, no formal sample-size or power calculation was performed, as this retrospective analysis was exploratory in nature. Accordingly, the present findings should be interpreted with caution and regarded as hypothesis-generating rather than definitive.

## Conclusions

The incidence of anastomotic leakage after TaTME in our cohort was comparable to rates reported for other rectal cancer surgical techniques. Leakage was independently associated with anastomotic technique, while tumour height emerged as a key determinant influencing both technique selection and leakage risk. Overall, TaTME appears to be a feasible option for selected patients in experienced centres; however, further validation in larger multicentre cohorts is required to strengthen the evidence base and to inform optimal anastomotic technique selection.

## Data Availability

The datasets generated and/or analyzed during the current study are available from the corresponding author on reasonable request.
